# Missed nursing care in acute care hospital settings in low-middle income countries: a systematic review protocol

**DOI:** 10.12688/wellcomeopenres.17431.2

**Published:** 2022-04-05

**Authors:** Abdulazeez Imam, Sopuruchukwu Obiesie, Jalemba Aluvaala, Michuki Maina, David Gathara, Mike English

**Affiliations:** 1 Oxford Centre for Global Health Research, Nuffield Department of Medicine, University of Oxford, Oxford, OX1 3SY, UK; 2 Independent Researcher, Asaba, Delta, Nigeria; 3 KEMRI-Wellcome Trust Research Programme, Nairobi, Kenya; 4 Department of Paediatrics, University of Nairobi, Nairobi, Kenya; 5 MARCH Centre, London School of Hygiene and Tropical Medicine, London, UK

**Keywords:** Implicit rationing, Task incompletion, Unmet nursing needs, developing countries, quality of patient care, Omission of nursing care

## Abstract

**Background:**  Missed nursing care (care left undone or task incompletion) is viewed as an important early predictor of adverse patient care outcomes and is a useful indicator to determine the quality of patient care. Available systematic reviews on missed nursing care are based mainly on primary studies from developed countries, and there is limited evidence on missed nursing care from low-middle income countries (LMICs). We propose conducting a systematic review to identify the magnitude of missed nursing care and document factors and reasons associated with this phenomenon in LMIC settings.

**Methods and analysis:**  This protocol was developed using the Preferred Reporting Items for Systematic Reviews and Meta-analysis Protocols (PRISMA-P). We will conduct literature searching across the Ovid Medline, Embase and EBSCO Cumulative Index to Nursing and Allied Health Literature (CINAHL) databases, from inception to 2021. Two independent reviewers will conduct searches and data abstraction, and discordance will be handled by discussion between both parties. The risk of bias of the individual studies will be determined using the Newcastle-Ottawa Scale (NOS).

**Ethics and dissemination**: Ethical permission is not required for this review as we will make use of already published data. We aim to publish the findings of our review in peer-reviewed journals

**PROSPERO registration number: **CRD42021286897 (27 ^th^ October 2021)

## Introduction

Missed nursing care is an umbrella term that describes nursing care that is either partially or completely omitted or delayed. It encompasses all aspects of nursing care including clinical, emotional care and administrative nursing duties ^1^. It has been described by many terms in literature including ‘task incompletion’, ‘unmet needs’ or ‘implicit rationing’ ^2^. It largely arises from an implicit prioritisation of some tasks at the expense of others and is due largely to competing demands for nursing time ^3^. While missed nursing care, in theory, might occur in all care settings where nurses play a role, the current evidence for this phenomenon is almost exclusively described in acute care hospital settings ^2^ Missed nursing care has gained much significance in nursing literature and practice from hospital settings where it is viewed as an early precursor and mediator for adverse patient health outcomes and an early signal for deteriorating quality of care ^2, 4^. Some studies have demonstrated associations between missed nursing care and negative patient care outcomes, for example medication errors, patient falls, nosocomial infections, pressure ulcers, increases in the risk of readmission following discharge, mortality and decreased patient satisfaction ^1, 4– 7^.

Despite the importance of the identification of missed nursing care, evidence for this phenomenon has come largely from high income countries (HICs) ^2, 4, 8^. Pre-review, we identified two recently published systematic reviews on missed nursing care that do not report any findings from low-middle income countries (LMICs) ^1, 2^. We also identified a few recent studies that investigated missed nursing care in LMIC contexts ^9– 11^. Generally, LMIC settings have distinctively different hospital structures, practice environments and organisational contexts from HICs and also have limited resources including staff and technology. It is thus conceivable that the magnitude, categories of most frequently missed nursing care and its associated factors might differ significantly from those of more developed settings.

To address the aforementioned gaps in evidence, we propose a systematic review to document the magnitude (how much care is missed), categories of most frequently missed nursing care and their associated factors and reasons in LMIC contexts. Our review builds on previous LMIC focused systematic reviews on nursing staffing and patient outcomes which have not included missed nursing care ^12^. It will also be important to guide the conduct of future nurse staffing research in LMICs and provide important information for policymakers.

### Objective and questions

This review will have four objectives:

1. Document the magnitude of missed nursing care in LMICs. and
2. Identify the categories of nursing care (specific nursing tasks) that are most frequently missed in acute hospital settings in LMICs.
3. Document the factors associated with missed nursing care in LMIC settings.
4. Document the reasons associated with missed nursing care in LMICs

## Protocol

The protocol for this systematic review was developed using the Preferred Reporting Items for Systematic Reviews and Meta-analysis Protocols (PRISMA-P) ^13^ and a completed PRISMA-P checklist is available in the *Extended data* ^14^. Our review was also registered with the International Prospective Register of Systematic Reviews (PROSPERO) on 27 ^th^ October 2021 (Registration number - CRD42021286897)

### Eligibility criteria

***Study design*.** Our systematic review will focus on missed nursing care, an important patient care outcome that has not previously been the focus of published reviews on nurse staffing in LMICs ^12, 15^. It will review both observational and interventional studies that describe or investigate missed nursing care in LMIC settings. The broad range of study types included will allow us to review missed nursing care in LMICs on a wider scale. We will however exclude qualitative, mixed-method studies, as our focus is more quantitative (identifying the magnitude and risk factors of missed nursing care). We will also exclude research that does not make use of primary data, for example other systematic reviews, umbrella reviews, protocols, and commentaries. The world bank country and lending group classification system will be used to identify LMICs. This system divides countries into low-income, low-middle-income and upper-middle-income economies based on gross national income per capita.

***Population*.** We will include all original studies that primarily focus on missed nursing care among patients admitted to LMIC hospital settings at all levels of care. To broadly describe the magnitude and types of care missed in hospitals, we will place no restrictions on the type of hospital wards where the study populations were recruited. We will thus consider regular staffed wards, for example medical, surgical or paediatrics, and wards with enhanced staffing such as intensive care wards. We will, however, exclude studies where the patient population were recruited in ambulatory care, for example immunisation or out-patient clinics, as care provided in such settings are distinctly different from in-patient care which is the focus of this review. For multi-country studies conducted across both HIC and LMIC settings, we will include these if the authors report their LMIC results separately.

***Exposures*.** Our exposure for this review will be the categories, reasons and risk factors associated with missed nursing care described in primary research. In this review, risk factors will be patient, nurse or hospital level variables that have been shown to be associated with missed nursing care, while reasons will be rationales put forward by nurses as to why missed nursing care occurs. We can quantitatively abstract reasons for missed nursing care from primary research as one of the main missed nursing care tools, the MISSCARE survey tool has a structured section for collecting data on pre-specified reasons for missed nursing care such as reduced staffing or unavailability of essential medical equipment ^17^. Some published studies using the MISSACRE tool have asked nurses to rank the reasons for the care that was missed during their previous shifts ^10, 18^.

Multiple missed nursing care tools describe different categories of nursing care/tasks ^9, 19– 21^, but it is feasible to thematically group this into themes or domains. Kalisch *et al.* describe nine themes for nursing care that is missed i.e., patient ambulation, turning, feedings, patient teaching, discharge planning, emotional support, hygiene, intake and output documentation, and surveillance ^17^. We propose to use this categorisation or, depending on the results of the review, use a more appropriate categorisation.

### Outcome

Our outcome for this review is the magnitude of missed nursing care. Studies on missed nursing care either report an overall percentage of care that was missed or a summary Likert score ^22, 23^. For the purposes of this review, we will document the magnitude of care missed across studies by documenting the range of overall percentages of care missed across studies.

Missed nursing care has been investigated using other synonyms, for example, omission of care, unmet nursing needs and implicit rationing of nursing care ^24^. For the current review, we will summarise all LMIC studies on missed nursing care irrespective of the methods or missed nursing care synonyms used. For studies to be eligible for inclusion in our review they should either report on one, or any combination of, the following categories: categories, magnitude of nursing care that are missed and factors and reasons associated with missed nursing care in LMIC settings. If it is feasible to extract specific data on missed nursing care, we will also include studies where missed nursing care is not the main variable of the study (for example, studies that report on missed nursing care together with multiple other patient care) 

We will exclude studies that examine missed care among other cadres of healthcare professionals. Studies reporting medication errors among nurses will also be excluded, as these do not represent omitted nursing tasks but occur largely due to acts of commission. We will also exclude papers not published in the English language due to limitations in translation.

### Search strategy

We will perform initial searches in Prospero to identify any ongoing or planned reviews that relate to our proposed research before undertaking the review. We contacted a health information librarian to develop our search strategy, and this was piloted in Medline (see *Extended data* ^14^). We will conduct additional searches in Embase, Cumulative Index to Nursing and Allied Health Literature (CINAHL), Global Health. No publication date restriction filters will be applied to our searches. Following primary database search, we will conduct additional literature through hand searching in select journals and forward-searching in Scopus. We will also check the references of published systematic reviews on missed nursing care to identify relevant primary articles.

### Data management

We will upload our search output into the Zotero reference management software, where we will perform initial de-duplication, and then utilise Microsoft Excel for the second round of de-duplication. We will then screen the titles and abstracts of our search output using the Rayyan – Intelligent Systematic Review software, a web-based application for screening ^25^. This will be performed independently by two reviewers (AI and SO) to select a set of potentially relevant articles, following which both reviewers will deliberate on a final set of articles for full-text screening. Disagreements will be resolved through discussions, if this is not successful, a third reviewer will serve as an arbitrator.

### Data items

We will develop a standardised Microsoft Excel form to abstract data from our identified primary articles. This will include, as a minimum, the publication year, name of the first author, country, research context, type of care that is missed, how missed care was measured and factors and reasons for care that were missed. Both reviewers will independently abstract information from the selected primary articles. Any disagreements will be resolved through discussion and, if necessary, a third reviewer might serve as an arbitrator for unresolved conflicts.

### Assessment of study quality

For studies that meet our eligibility criteria, we will employ the Newcastle-Ottawa Scale (NOS) ^26^. This scale widely used to appraise the quality of non-randomised studies ^26^. There is also a published adaptation of this tool for cross-sectional studies ^27^. This consists of three domains:

a. Selection which appraises the representativeness of the study sample, its size and how valid the measurement of exposure is.

b. Comparability of the study groups.

c. validity of the outcome measure. 

Typically, each of the 3 domains sum up to a maximum 10 and researchers might either pre-specify a value as a cut-off to include a study or not. For this review, we will not include a cut-off because we aim to provide broad information on missed nursing care in LMICs. We will include all eligible studies in our synthesis irrespective of their risk of bias scores, but we will discuss any potential impact of these scores in our evidence synthesis

We have selected the NOS as we anticipate we are unlikely to find any intervention studies or randomised control trials for missed care in LMICs through our search. Recent reviews on missed nursing care did not report any intervention research or randomised controlled trials ^1, 2^.

The risk of bias assessment will be conducted independently by two reviewers and any differences will be addressed by discussion. A third reviewer will be called to review any unresolved conflicts. 

### Data synthesis

We will consider pooling data and where not feasible, resort to a narrative synthesis. This is because primary studies of missed nursing care are heterogeneous in terms of the methods and tools used to measure missed nursing care and how they present their results. Missed nursing care has been measured using either direct observational methods or subjective patient and nurse reporting of care that is missed ^1, 8, 9^. Results of studies on missed nursing care are also frequently presented in different formats, as either the proportion of care that is missed or as a mean/median score when a Likert scale is used ^22, 28, 29^. To describe the most frequently missed categories of missed nursing care, we will report either of these estimates and rank order them to identify the three most and least frequently missed nursing care categories.

### Ethics and dissemination

Our review is secondary research and so will not require any ethical approval. We aim to publish our findings in a peer-reviewed journal.

### Study status

We confirm that by the time of submission of this protocol we have completed our search and are conducting full-text screening of identified articles.

## Discussion

Donabedian described the structure-process-outcome framework, which has been a cornerstone in describing and researching the quality of patient care ^30^. In summary, structures or the setup of a health system affect care processes which in turn are likely to affect health care outcomes ^31^. Missed nursing care is a process-based indicator of the quality of patient care and is likely to signal deterioration in patient care ahead of traditional outcome-based quality indicators such as mortality or length of stay. It is also possible missed nursing care might show earlier responses to interventions aimed at improving the quality of patient care compared to outcome-based quality indicators, underscoring the importance of this indicator. Traditionally, literature on missed nursing care has primarily been from more developed settings since the term was first described by Kalisch *et al.* ^17^. In the last two to three years, there have been increasing missed nursing care publications from LMIC settings ^9, 10, 29^, these are not currently reflected in the most recent reviews ^1, 2^. Our systematic review will sum up the current evidence on missed nursing care in LMIC settings.

A recurring theme in missed nursing care literature is the inverse relationship between nurse staffing levels and the magnitude of missed nursing care ^2, 9^. Traditionally, LMICs have poorer nurse staffing in comparison to HICs and a higher magnitude of missed nursing care described in LMICs would have implication for nurse staffing policies, suggesting a need for urgent improvement in nurse staffing levels. The challenge in many of these settings is financing Human Resources for Health which although a global problem is more prevalent in resource constrained LMICs. There is however an opportunity if the most frequently missed categories of nursing care are non-clinical duties (for example, provision of physical needs) as this might provide a justification for hiring lower skilled nurse support workers or assistants in settings where they are non-existent. This cadre of staff are paid less than nurses and are a recognised part of the health workforce in more developed climes ^32^. If such policies are implemented, nursing assistants should not substitute existing nurses but rather be supplementary to them as there is evidence from HICs suggesting substitution of nurses might have a negative effect on care quality ^33^.

## Data availability

### Underlying data

No underlying data are associated with this article

### Extended data

Open Science Framework: Missed nursing care in acute care hospital settings in low-middle income countries: a systematic review protocol. https://doi.org/10.17605/OSF.IO/JZXRV ^13^

This project contains the following extended data:

- Medline search strategy.docx (Medline search strategy for this proposal)

### Reporting guidelines

Open Science Framework: PRISMA-P checklist for ‘Missed nursing care in acute care hospital settings in low-middle income countries: a systematic review protocol’. https://doi.org/10.17605/OSF.IO/JZXRV ^13^

Data are available under the terms of the Creative Commons Attribution 4.0 International license (CC-BY 4.0).
