# Supplementary material for: Missed nursing care in acute care hospital settings in low-middle income countries: a systematic review protocol
Source: Wellcome Open Res. 2022 Apr 5;6:359. Originally published 2021 Dec 21. [Version 2] doi: 10.12688/wellcomeopenres.17431.2 (PMC9020529; doi:10.12688/wellcomeopenres.17431.2)
Supplement: Peer review history

# Peer review: Reviewer response for version 2

**DOI:** 10.21956/wellcomeopenres.19713.r49761

**Published:** 2022-04-19

**Authors:** Joseph Okebe

**Affiliations:** 1 Department of International Public Health, Liverpool School of Tropical Medicine, Liverpool, UK

I am satisfied with the response from the authors.

Is the study design appropriate for the research question?

Yes

Is the rationale for, and objectives of, the study clearly described?

Partly

Are sufficient details of the methods provided to allow replication by others?

Partly

Are the datasets clearly presented in a useable and accessible format?

Not applicable

Reviewer Expertise:

Research methods, paediatrics

I confirm that I have read this submission and believe that I have an appropriate level of expertise to confirm that it is of an acceptable scientific standard.

# Peer review: Reviewer response for version 2

**DOI:** 10.21956/wellcomeopenres.19713.r49762

**Published:** 2022-04-06

**Authors:** Marica Cassarino

**Affiliations:** 1 School of Applied Psychology, University College Cork, Cork, Ireland

I thank the authors for working on the recommended revisions. All my comments have been addressed and I have no further points to raise. I wish the authors all the best with this review.

Is the study design appropriate for the research question?

Yes

Is the rationale for, and objectives of, the study clearly described?

Yes

Are sufficient details of the methods provided to allow replication by others?

Partly

Are the datasets clearly presented in a useable and accessible format?

Not applicable

Reviewer Expertise:

Applied Psychology; Health Services Research

I confirm that I have read this submission and believe that I have an appropriate level of expertise to confirm that it is of an acceptable scientific standard.

# Peer review: Reviewer response for version 1

**DOI:** 10.21956/wellcomeopenres.19274.r48768

**Published:** 2022-02-18

**Authors:** Marica Cassarino

**Affiliations:** 1 School of Applied Psychology, University College Cork, Cork, Ireland

The manuscript presents a protocol for a systematic review of missed nursing care in low-medium income settings (LMIS) and the factors associated with missed nursing care. The authors state that the rationale for this review derives from a lack of evidence synthesis on the topic in LMIS and the main focus on acute settings in the available literature. The protocol is in line with the PROSPERO registration. The review methods are overall clear, although some points need clarification. I have listed my comments below: 

1. Study objectives: It is unclear what the authors mean by the "magnitude" of nursing care. There is a justification of categories, but the definition of magnitude is needed. Does this entail frequency?
2. The Introduction states that there is a gap in the literature in that most evidence on the topic focuses on acute settings, which entails that this review will look more broadly at different types of care settings. However, objective 1 states that the review will focus on acute hospital settings. This needs clarification.
3. Linked to comment 2, I would encourage the authors to clarify why ambulatory care or out-patient clinics are outside the scope of the review. 
4. Study design: The authors state that systematic reviews will be excluded. However, I would expect that existing reviews should be checked to see if any primary studies are relevant to this review. It would be useful to state the same. 
5. Search strategy: Please state if any publication date filters have been used. 
6. Data management: This may not be relevant as the authors state that they are already screening full-texts, but just as a piece of advice, Rayyan manages duplicates with quite high accuracy, without using other software.
7. Outcome: Should studies be included if missed nursing care is not the primary outcome but it is possible to extrapolate relevant information?

Is the study design appropriate for the research question?

Yes

Is the rationale for, and objectives of, the study clearly described?

Yes

Are sufficient details of the methods provided to allow replication by others?

Partly

Are the datasets clearly presented in a useable and accessible format?

Not applicable

Reviewer Expertise:

Applied Psychology; Health Services Research

I confirm that I have read this submission and believe that I have an appropriate level of expertise to confirm that it is of an acceptable scientific standard, however I have significant reservations, as outlined above.

# Response

**Published:** 2022-03-22

**Authors:** Abdulazeez Imam

**Affiliations:** University of Oxford, UK

We thank the reviewer for making time to read our manuscript and providing invaluable feedback. We have responded to all their comments and our responses come in italics under each specific comment

 Comment 1: Study objectives: It is unclear what the authors mean by the "magnitude" of nursing care. There is a justification of categories, but the definition of magnitude is needed. Does this entail frequency?

*Response:*

*We thank the reviewer for this comment and have now defined magnitude. Please see the introduction, line 28.*

 Comment 2: The Introduction states that there is a gap in the literature in that most evidence on the topic focuses on acute settings, which entails that this review will look more broadly at different types of care settings. However, objective 1 states that the review will focus on acute hospital settings. This needs clarification.

*Response:*

*We thank the reviewer for this comment. Stating the evidence was mostly from acute care settings was a justification for our review to focus on these settings and not to address a gap. We have re-organised our introduction to reflect this. Please see the introduction, lines 7 to 16.*

 Comment 3: Linked to comment 2, I would encourage the authors to clarify why ambulatory care or out-patient clinics are outside the scope of the review. 

*Response:*

*We thank the reviewer for this comment. We have clarified this, please see population, lines 64 to 67.* 

 Comment 4: Study design: The authors state that systematic reviews will be excluded. However, I would expect that existing reviews should be checked to see if any primary studies are relevant to this review. It would be useful to state the same.

*Response:*

*We thank the reviewer for this comment. We have modified the protocol to include this statement. Please see search strategy, lines 112 to 115.*

 Comment 5: Search strategy: Please state if any publication date filters have been used.

*Response:*

*We will not use any date restriction filters. Please see search strategy, lines 111 to 112.*

**

 Comment 6: Data management: This may not be relevant as the authors state that they are already screening full texts, but just as a piece of advice, Rayyan manages duplicates with quite high accuracy, without using other software.

*Response:*

*We thank the reviewer for this comment.*

 Comment 7: Outcome: Should studies be included if missed nursing care is not the primary outcome, but it is possible to extrapolate relevant information?

*Response:*

*We thank the reviewer for this comment, and we have now modified our protocol to include such papers* *. Please see outcome, lines 97 to 100.*

# Peer review: Reviewer response for version 1

**DOI:** 10.21956/wellcomeopenres.19274.r47754

**Published:** 2022-01-17

**Authors:** Joseph Okebe

**Affiliations:** 1 Department of International Public Health, Liverpool School of Tropical Medicine, Liverpool, UK

This is a very interesting review proposal and the authors have made a strong case for the review going forward.

 I have a few comments that could improve the review:

- Consistency in the use of terms: "categories, levels, types, magnitude" were used interchangeably without clarity on what they mean.
- Objectives were rather complex: You want to identify " *magnitude and categorie*s" and, document " *factors associated with and reasons*". These are different concepts. Consider defining one per objective or streamline.
- Protocol: Consider how magnitude will be coded/graded since the review is quantitative. It appears you are referring to the themes described by Kalisch *et al.* ^1^. If so, consider using similar terms for consistency.
- Protocol: I am also not sure what the difference is between " *associated risk factors and reasons for*". I feel association is a plausible outcome to be measured than reasons for.
- Outcome: How will magnitude be measured/coded? Also, I  suggest reconsidering the choice of excluding studies because missed nursing care is not the main variable reported in a study. If there is data on missed care, it should be captured unless it is presented in such a way that precludes extraction.
- Data synthesis: I suggest considering pooling data and where not feasible, resort to a narrative synthesis.
- Quality assessment: I know the Newcastle-Ottawa score is popular, but it may be useful to include a summary of either the variables or the scoring for readers who are not used to it.
- Discussion: I would like to see some reflections on the implications of the findings for policy, research, and nurse training.
- The concept analysis paper by Kalisch *et al.* ^2^ is quite a useful read. The paper provides a detailed theoretical background to the concept of missed nursing care that would be useful in describing and contextualising the findings of the review. I found it quite useful to get a grasp of what the review is trying to do. I note that it was not referenced in the paper and felt this is a useful additional resource in the background and perhaps discussion.

Overall, a clear presentation of work. Well done to the authors.

Is the study design appropriate for the research question?

Yes

Is the rationale for, and objectives of, the study clearly described?

Partly

Are sufficient details of the methods provided to allow replication by others?

Partly

Are the datasets clearly presented in a useable and accessible format?

Not applicable

Reviewer Expertise:

Research methods, paediatrics

I confirm that I have read this submission and believe that I have an appropriate level of expertise to confirm that it is of an acceptable scientific standard, however I have significant reservations, as outlined above.

## References

[1] : Missed nursing care: a qualitative study. *J Nurs Care Qual* .21(4) : 10.1097/00001786-200610000-00006 306-13; quiz 314 10.1097/00001786-200610000-00006 16985399

[2] : Missed nursing care: a concept analysis. *J Adv Nurs* .2009;65(7) : 10.1111/j.1365-2648.2009.05027.x 1509-17 10.1111/j.1365-2648.2009.05027.x 19456994

# Response

**Published:** 2022-03-22

**Authors:** Abdulazeez Imam

**Affiliations:** University of Oxford, UK

We thank the reviewer for making time to read our manuscript and providing invaluable feedback. We have responded to all their comments and our responses come in italics under each specific comment.

 Comment 1: Consistency in the use of terms: "categories, levels, types, magnitude" were used interchangeably without clarity on what they mean

*Response:*

*Thank you for this comment. We have now streamlined the use of these terms throughout the manuscript and stuck to two terms, magnitude and categories. We have also defined both terms. Please see the introduction, line 28 and objective 2, lines 36-37.*

 Comment 2: Objectives were rather complex: You want to identify "magnitude and categories" and, document "factors associated with and reasons". These are different concepts. Consider defining one per objective or streamline.

*Response:*

*We thank the reviewer for this comment, and we have now split the objectives of the review into 4. Please see Objective and questions, lines 35-39.*

 Comment 3: Protocol: Consider how magnitude will be coded/graded since the review is quantitative. It appears you are referring to the themes described by Kalisch *et al.* ^1^ . If so, consider using similar terms for consistency

*Response:*

*We thank the reviewer for this comment. The magnitude will be determined from the individual papers by extracting the overall proportion of care that is missed. Please see line outcome, lines 86-90. The Kalisch classification or a more appropriate classification will be used to identify categories of care thematically, please see Exposures, lines 79-84.*

 Comment 4: Protocol: I am also not sure what the difference is between " *associated risk factors and reasons for*". I feel association is a plausible outcome to be measured than reasons for.

*Response:*

*We have now clarified this better and how we would abstract reasons. Please see Exposures, lines 69-78*

 Comment 5: Outcome: How will magnitude be measured/coded? Also, I  suggest reconsidering the choice of excluding studies because missed nursing care is not the main variable reported in a study. If there is data on missed care, it should be captured unless it is presented in such a way that precludes extraction.

*Response:*

*We thank the reviewer for this comment. Magnitude will be determined from the individual papers by extracting the overall proportion of care that is missed. Please see outcome, lines 86-90.*

*We understand the reviewer's concern about excluding studies where missed nursing care is not the main variable reported and have now revised our protocol to include this. Please see outcome, lines 97-100.*

 Comment 6:  Data synthesis: I suggest considering pooling data and where not feasible, resort to a narrative synthesis.

*Response: This has now been modified. Please see Data synthesis, lines 155-159*

 Comment 7: Quality assessment: I know the Newcastle-Ottawa score is popular, but it may be useful to include a summary of either the variables or the scoring for readers who are not used to it.

*Response:*

*We thank the reviewer for this comment and we have now provided summary information for the Newcastle-Ottawa score. Please see Assessment of study quality, lines 133-146.*

 Comment 8: Discussion: I would like to see some reflections on the implications of the findings for policy, research, and nurse training.

*Response:*

*We thank the reviewer for this comment and have provided our reflections in the discussion, lines 185-198.*

**

 Comment 9: The concept analysis paper by Kalisch et al. ^2^ is quite a useful read. The paper provides a detailed theoretical background to the concept of missed nursing care that would be useful in describing and contextualising the findings of the review. I found it quite useful to get a grasp of what the review is trying to do. I note that it was not referenced in the paper and felt this is a useful additional resource in the background and perhaps discussion.

*Response:*

*The reviewer is right about this landmark paper by Kalisch who coined the term missed nursing care. We have drawn from this paper to inform our introduction. Please see the introduction, lines 5-7. We have also drawn on other works by Kalisch throughout the manuscript.*
